# Identifying parameters to improve the reproducibility of transient gene expression in High Five cells

**DOI:** 10.1371/journal.pone.0217878

**Published:** 2019-06-06

**Authors:** Maren Bleckmann, Margitta Schürig, Michelle Endres, Anke Samuels, Daniela Gebauer, Nadine Konisch, Joop van den Heuvel

**Affiliations:** 1 Department Recombinant Protein Expression Facility, Rudolf Virchow Centre, Würzburg, Bavaria, Germany; 2 Department Recombinant Protein Expression, Helmholtz Centre for Infection Research, Braunschweig, Lower Saxony, Germany; Universite Claude Bernard Lyon 1, FRANCE

## Abstract

Virus-free, transient gene expression (TGE) in High Five cells was recently presented as an efficient protein production method. However, published TGE protocols have not been standardized to a general protocol. Therefore, reproducibility and implementation of the method in other labs remains difficult. The aim of this study is to analyse the parameters determining the reproducibility of the TGE in insect cells. Here, we identified that using linear 40 kDa PEI instead of 25 kDa PEI was one of the most important aspects to improve TGE. Furthermore, DNA amount, DNA:PEI ratio, growth phase of the cells before transfection, passage number, the origin of the High-Five cell isolates and the type of cultivation medium were considered. Interestingly, a correlation of the passage number to the DNA content of single cells (ploidy) and to the transfection efficacy could be shown. The optimal conditions for critical parameters were used to establish a robust TGE method. Finally, we compared the achieved product yields in High Five cells using our improved TGE method with both the baculoviral expression system and TGE in the mammalian HEK293-6E cell line. In conclusion, the presented robust TGE protocol in High Five cells is easy to establish and produces ample amounts of high-quality recombinant protein, bridging the gap in expression level of this method to the well-established mammalian TGE in HEK293 cells as well as to the baculoviral expression vector system (BEVS).

## Introduction

Protein expression in insect cells has serval advantages. Insect cell lines offer the majority of posttranslational modifications e. g. phosphorylation of serine [1], tyrosine sulfation [2] or palmitoylation [3], while being easier to handle than mammalian cells. In comparison to mammalian cells, insect cells can be cultivated without additional CO_2_ aeration at 27°C in serum-free media, thereby decreasing costs for the experimental set-up and reducing the risk of contamination with human pathogens [4]. However, compared to mammalian cells the glycosylation pattern in insect cells mainly consists of truncated N-glycans of the paucimannose type [5]. This glycosylation pattern is a relevant difference for secreted glycoprotein from insect cells, as mammalian glycosylation might be required for pharmaceutical applications and/or full biologic activity. To circumvent this limitation, both insect cell lines and baculovirus expression system have been adapted to achieve authentic mammalian-like glycosylation [6–9]. On the other side, the paucimannose glycosylation type is more homogenous and less flexible, which presents an advantage for 3 D structural analysis by crystallization. The high number of eukaryotic membrane protein structures deposited in the Protein Database (PDB) reflects this advantage of proteins produced in insect cells [10].

For many years the baculovirus expression vector system (BEVS) has been successfully employed to produce large amounts of recombinant protein in insect cells [11–13]. The BEVS was further improved over time to more easily and faster generate recombinant baculovirus [14], by the elimination of viral protease genes [15], achieving higher viability during expression [16] and the option to insert multiple expression cassette into one vector-backbone [17]. In general, BEVS is considered safe as baculovirus cannot replicate in humans [18]. Especially the EMA and FDA approval for pharmaceutical applications for production of vaccines in insect cells is very promising [19], as it allows a quick reaction to unforeseen pandemics, much faster than traditional vaccine production using eggs or stable cell lines [20].

Nevertheless, BEVS is still a time-consuming, material and labour-intensive technology compared to TGE. Particularly, the generation of high-quality virus takes time despite recently improved protocols for faster baculovirus generation [14]. Besides, long-time storage of baculovirus still remains cumbersome in spite of efforts to develop new storage methods [21, 22]. Furthermore, intrinsic genetic recombination within the baculoviral genome may lead to excision of the gene of interest during multiple rounds of virus generation [23, 24]. In addition, the viral infection process interferes with the cellular integrity and may influence the quality of the target proteins by hampering the host cell protein production and secretion machinery [25]. Especially the quality of secreted target proteins is impaired, using the strong late viral p10 and polH promoters for expression [26]. Furthermore, virus-free expression for the production of secreted virus-like particles is favoured, because the plasmid-based TGE avoids contamination of the samples with baculoviral particles [27], which are hard to separate afterwards [28]. Another advantage of TGE compared to BEVS is the possibility of doing co-transfection for the production of protein complexes without the need of complex multicistronic expression vectors [17].

In order to improve the throughput and sensitivity of detection of successful expression constructs, fast screening strategies using TGE combined with transactivation [29, 30] or the SplitGFP technology [31] have been established. These methods avoid the need to generate high amounts of different recombinant baculoviruses. In this way, proper construct screening reduces time and effort to detect expressible constructs before going to the final scale-up and production using BEVS. Still, a complete abandonment of the viral infection before entering the production phase would be even more beneficial. Therefore, plasmid-based transient protein expression using Polyethlyenimine (PEI) has been recently established [32–35]. However, the described transfection protocols are difficult to compare and so far rather challenging to implement in different laboratories.

Therefore, we analyse in this study the different parameters determining the reproducibility of the TGE in High Five cells. Firstly, we compare the published methods for PEI transfection. Secondly, we investigate the factors improving the reproducibility of the method. Thereafter, we show a simple, reliable and reproducible PEI transfection protocol using PEI 40 kDa allowing for high yield expression of recombinant protein. Finally, we underline the applicability of the TGE method in High Five cells by comparing the achieved protein yields to the production level using TGE in HEK293-6E cells [36] or BEVS.

## Materials and methods

### Expression plasmids

Suitable expression plasmids were chosen for the respective expression system. The transient transfection in High Five cells was facilitated with the pOpiE2 plasmid, which is in our hands the best expression vector for virus-free protein expression [37]. Important to note is that it does not only contain the immediate early promoter OpiE2 but also the highly functional IE1 terminator, as well as enhancing sequences [30]. The Multi-Host vector pFlpBtM-II was used for expression in HEK293-6E cells and for generating the EmBacY baculoviral genome for BEVS [38]. The target genes were cloned into the respective multi-cloning sites using either restriction digestion according to standard protocols or by sequence and ligation independent cloning (SLIC) [39]. The plasmids used are named pOpiE2-eGFP-HA; pFlpBtM-II-H1, pOpiE2-H1 and pcDNA3 (stuffer DNA vector). Every expression plasmid was prepared by the NucleoBond PC, Xtra (Macherey-Nagel) or the PureYield (Promega) Midiprep Kit according to the description of the manufacturer. DNA purification kits from other manufacturer like the endotoxin free plasmid purification kit of Qiagen equally well-suited to generate high quality DNA samples for TGE.

### Cell culture

BTI-Tn-5B1-4 (*Trichioplusia ni*, High Five) or Sf21 (*Spodoptera frugiperda*, DSMZ #ACC119) cells were maintained in exponential growth and passaged every 2–3 days to 0.3–0.5x10^6^ cells/mL. If not otherwise mentioned, High Five cells were derived from an early clonal isolate of the Boyce-Thompson Institute and were cultivated in suspension in EX-CELL 405 medium (Sigma-Aldrich) and Sf21 cells in EX-CELL 420 medium (Sigma-Aldrich) at 27°C and shaking at 130 rpm (Kuhner, 50 mm orbit). Vent Cap Polycarbonate Corning Erlenmeyer flasks (125 mL vessel filled with 20–40 mL medium or 500 mL vessel filled with 120–200 mL medium) were used for backup culture and protein production. Small scale experiments were performed in 50 mL tubes (Sarstedt) filled with 1–6 mL volume and shaking at 180–200 rpm (Kuhner, 25 mm orbit).

Other High Five cell line isolates were obtained from Thermo Fisher Scientific- further on named TFS-1 (BTI-TN-5B1-4, catalogue number B85502) and the same cell line was additionally kindly provided by Puente-Massaguer (Universitat Autònoma de Barcelona) after adaption to Insect Xpress medium and another adaption to Sf-900 III SFM medium- further on named TFS-2 (Thermo Fisher Scientific). Next to EX-CELL 405 medium, Sf-900 II SFM and Sf-900 III SFM medium (Thermo Fisher Scientific) was used to cultivate the High Five cells. The specific growth rate μ of the High Five cells in the different media ranges from 0.040 to 0.045 h^-1^ with an average doubling time of 15 to 16 h. The exponential growth phase for High Five cells was between 0.4 to 4.0 x 10^6^ cells/ml.

### PEI batches used in this study

PEI was supplied by Polysciences. The linear 25 kDa PEI lot numbers #579115 (PEI 25 kDa Lot#1) and #696676 (PEI 25 kDa Lot#2) and the linear 40 kDa PEI lot numbers #697970 (PEI 40 kDa Lot#1) and #706671 (PEI 40 kDa Lot#2) were used in this study. If not otherwise indicated PEI 40 kDa Lot#1 was used in the experiments. Linear 25 kDa PEI was solved in deionized water either by decreasing the pH to pH 2 or heating. Linear 40 kDa PEI is soluble in deionized water at RT. If not otherwise indicated the PEI solution was adjusted to pH 7 and sterilized by filtration. Linear 25 kDa PEI was kept at - 80°C for long-term and at– 20°C for short-term storage. Linear 40 kDa PEI was stored at 4°C.

### PEI transfection protocols

#### Adaptation of the Shen *et al*. protocol [33]

High Five cells were passaged to 0.5x10^6^ cells/mL 24 h before transfection. For transfection 4x10^6^ cells were centrifuged (180 xg, RT, 4 min) and resuspended in 2 mL fresh EX-CELL 405 medium (total cell density = 2x10^6^ cells/mL). In total 4 μg DNA containing 5% pOpiE2-eGFP-HA plasmid and 95% stuffer DNA (pcDNA3, HEK expression plasmid) were added directly to the cells (1 μg DNA per 1x10^6^ cells/mL). Without delay 16 μg of a 1 mg/mL linear 25 kDa PEI solution (in H_2_O, pH 7) were also added directly to the cells (4 μg PEI per 1x10^6^ cells/mL). The cells were cultivated at 27°C and 180 rpm. After 24 h the cells were supplemented with 2 mL fresh medium to maintain exponential growth. Measurement of the eGFP took place 48 h after transfection using the cytometer (Cytoflex, Beckmann Coulter).

#### Adaptation of the Karste *et al*. protocol [34]

For transfection 4x10^6^ cells were centrifuged (180 xg, RT, 4 min) and resuspended in 1 mL fresh EX-CELL 405 medium (total cell density = 4x10^6^ cells/mL). In total 8 μg DNA containing 5% pOpiE2-eGFP-HA plasmid and 95% stuffer DNA (pcDNA3, HEK expression plasmid) were added directly to the cells (2 μg DNA per 1x10^6^ cells/mL). Without delay 32 μg of a 1 mg/mL linear 25 kDa PEI solution (in H_2_O, pH 7) were also added directly to the cells (8 μg PEI per 1x10^6^ cells/mL). The cells were cultivated at 27°C and 180 rpm. After the 3 h high cell density transfection step, the cells were supplemented with 3 mL fresh medium to obtain exponential growth. Measurement of the eGFP was done 48 h post transfection using the cytometer (Cytoflex, Beckmann Coulter; Guava easyCyte, merckmillipore).

#### Optimized PEI 40 kDa protocol

In comparison to Karste *et al*. the parameters were adjusted according to the results of the experiments described in this study. Thereafter, linear PEI 40 kDa was used instead of linear PEI 25 kDa and the required amount of DNA was reduced to 1 μg per 1x10^6^ cells/mL (95% of target DNA and 5% of pOpiE2-eGFP-HA plasmid). The DNA:PEI ratio was set to 1:4 and the cell density for the initial transfection reaction was set to 4x10^6^ cells/ml.

### PEI-DNA interaction studies

The PEI-DNA binding studies were performed according to Delafosse *et al*. [40]. The ability of PEI to condense DNA was investigated by analysing the reduction of the migration of the DNA in an agarose gel caused by PEI overlaying the negative charge of the DNA. Different DNA:PEI ratios were tested (1 μg plasmid DNA and 0 μg, 0.1 μg, 0.2 μg, 0.3 μg and 0.4 μg PEI). The complexes were formed 10 min at RT in water.

### Baculovirus expression

Baculovirus expression was done according the standard Bac-to-Bac protocols using the EmBacY [41] baculoviral genome. First, heat-shock competent DH10-EmBacY *E*. *coli* cells were transformed for 45 s at 42°C with the respective transfer vector (pFlpBtM-II-H1) and cultivated without addition of antibiotics for 4 h at 37°C and 600 rpm. The *E*. *coli* cells were than plated on an agar plate containing 50 μg/mL Kanamycin, 10 μg/mL Tetracycline, 7 μg/mL Gentamycin, 40 μg/mL IPTG and 100 μg/mL Bluo-gal. Blue-White selection was performed and white colonies were additionally analysed by PCR using *M13 for* (CCCAGTCACGACGTTGTAAAACG) and *M13 rev* (AGCGGATAACAATTTCACACAGG) primers. The PCR amplification was performed using Dream Taq DNA Polymerase (Thermo Fisher Scientific) according to the manufacturers protocol. The bacmid DNA was isolated using buffers of the DNA Midi-Kit (Promega) for cell lysis, protein precipitation and neutralisation. Afterwards the DNA was isolated using isopropanol precipitation. For each transfection 1.5 mL of 0.5x10^6^ Sf21 cells/mL in EX-CELL 420 medium containing 10% FCS were seeded in one well of a six well plate. Next 10 μL isolated bacmid (concentration ~500 ng/μL-2000 ng/μL) and 10 μL SuperFect (Qiagen) were diluted in 100 μL serum- free EX-CELL 420 medium and incubated 10 min at RT. After incubation 600 μL of serum-free medium was added to the bacmid-SuperFect complexes, which then were used to replace the medium of the adherent Sf21 cells. The transfection mixture was replaced after 2 h of incubation at 27°C with EX-CELL 420 medium containing 5% FCS and harvested five days post transfection. The viral stock solutions were generated by infecting 0.5x10^5^ Sf21 cells/mL with 10% of the previous Transfection Supernatant. The supernatants (viral amplificate VA) were harvested up to five days post infection. For determining the time of harvest, the viral infection was monitored by measuring the fraction of YFP positive cells using a cytometer (Guava easyCyte, merckmillipore), cell growth, viability and the increase in cell diameter (Casy Counter, Roche). Generally, three days after cell growth arrest, after reaching the maximal increase in cell diameter or upon a drop in viability of the cells below 80%, the supernatant was harvested.

### Analysis of the intracellular DNA content

The analysis was done as described in Jarman-Smith *et al*. [42]. Briefly, 5x10^6^ cells/mL were pelleted at 500 xg for 4 min. The pellet was resuspended in 20 mL cold 70% ethanol (- 20°C) and stored at - 18°C. At the day of the analysis the cell samples were centrifuged and the ethanol was removed. The pellet was washed once with PBS, pH 6.4. Afterwards the pellet was resuspended in 1 mL of RNase A solution (250 μg/mL in PBS, Merck) and incubated 20 min at 37°C. Finally, 50 μL Propidium Iodide (PI) (1 mg/mL in H_2_O) were added to each sample and incubated at least 5 min at RT before analysis in the cytometer (Cytoflex, Beckmann Coulter). The gates were adjusted to select single viable cells by doublet discrimination (PI area vs PI height).

### HA1 protein purification

His tagged HA1 (Hemagglutinin subunit 1, a surface protein of influenza virus) was purified directly from the supernatant using ÄKTA-FLPC (GE Healthcare) with HisTrap Excel prepacked columns (GE Healthcare). Prior to use, the columns were equilibrated according to the protocol of the manufacturer with 20 mM sodium phosphate, 0.5 M NaCl, pH 7.4. Subsequently, the filtered (0.45 μm) culture supernatant was loaded o/n using a sample pump with a flow rate of 50% of the column volume (CV) per minute. This was followed by a washing step of 20 CV with wash buffer containing 30–50 mM imidazole. Elution was performed with 250 mM imidazole for up to 10–20 CV. Analysis of protein expression and purification was performed by SDS-PAGE and Western blot analysis.

## Results and discussion

### Comparison of the published PEI transfection protocols

Shen *et al*. published 2015 the first efficient protocol for transient gene expression (TGE) of plasmid DNA in High Five cells using linear 25 kDa PEI [33]. This protocol could not be successfully established in our laboratory (HZI) as the high transfection rates shown by Shen *et al*. could not be reproduced. Therefore, we established and published a modified TGE protocol for our High Five cell line, which performed best in our hands (Karste et al., 2017). However, as we tried to transfer our technology to the new recombinant protein expression facility at the Rudolf-Virchow-Centre (RVZ), we observed again much lower transfection rates and viability. Comparison of the used materials clearly showed massive batch-to-batch variation of the 25 kDa PEI Lot#1 and Lot#2 (see Materials and Methods). The variation in performance of different linear 25 kDa PEI batches in TGE has been described before. This difference is most probably caused by residual propionylation that varies between individual batches from 4 up to 11% according to the manufacturer (Polysciences). In fact, some studies have shown an effect of both acetylation as well as deacetylation on transfection efficiencies for different mammalian cell lines [43–46]. The difference between 25 kDa PEI Lots impairs the reproducibility of the published protocols using linear 25 kDa PEI vastly and limits the transferability and application of the method in other laboratories.

Therefore, we searched for a robust alternative to linear 25 kDa PEI, with reproducible performance for each batch. The linear 40 kDa PEI (also known as PEI 22 kDa in free base or PEImax) was claimed to be an efficient alternative reagent for transient transfection. Most importantly, 40 kDa PEI is fully depropionylated, avoiding batch-to-batch variation in comparison to linear 25 kDa PEI. Indeed, using 40 kDa PEI for TGE in CHO and HEK cells performed even better than the traditionally used linear 25 kDa PEI [47].

**Fig 1:**
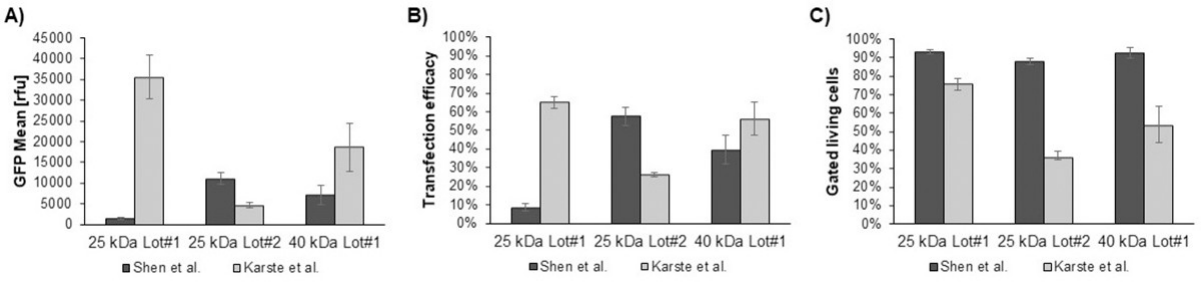
Comparison of the different protocols for PEI transfection. Different protocols using various linear PEI batches were compared as indicated. In total 5% OpiE2-eGFP plasmid and 95% stuffer DNA were used. A) GFP mean [rfu] B) percentage of transfected cells and C) the fraction/amount of gated living cells were determined by flow cytometry 48 hours post transfection. A cell line with over 140 passages in EXCELL-405 was used for these experiments. The data shown are the result of at least four independent experiments.

To compare different lots of 25 kDa and 40 kDa PEI for their performance in TGE in insect cells, the transfection agents were tested using both the protocol according to Shen *et al*. as well as according to Karste *et al*. (see Materials and Methods). Fig 1 shows a vast difference of the 25 kDa PEI Lots in the achieved eGFP yield ranging from 1.400 compared to 11.100 rfu (Shen *et al*.) and 4.600 compared to 35.600 rfu (Karste *et al*.). Obviously, the performance of the two 25 kDa PEI Lots is different in both protocols, e. g. the 25 kDa PEI Lot#1 achieved the best results with the Karste protocol whereas 25 kDa PEI Lot#2 performed better using the Shen protocol. Thus, for each new purchased 25 kDa PEI Lot, it will be required to optimize and adapt the protocol. Titration of the DNA:PEI ratio and optimization of the DNA concentration for each batch of 25 kDa PEI can rescue the transfection efficiency. The different PEI 25 kDa Lots were able to reach a similar transfection efficacy (60%, see Fig 1B) when used under their respective conditions. However, the GFP mean did not respond correspondingly (Fig 1A).

In addition to the transfection efficacy and the GFP mean, the vitality of the cells based on cell size and granularity was evaluated by flow cytometry to discover cytotoxic effects of the different PEI batches. The vitality of the culture however, cannot be equated directly to the fraction of gated living cells, as both dead cells as well as small cellular debris are excluded from counting. Still, the gated living cells represent the overall viable state of the cell culture. A lower overall number of cells per mL will negatively influence the overall volumetric recombinant protein yield, thus a high value of gated living cells is favoured during the experiments. In Fig 1, the fraction of gated living cells is drastically reduced when the 25 kDa PEI Lot#2 was used as described by Karste *et al*. In parallel the viability determined by microscopic cell counting in a Neubauer chamber showed a decrease to 55% living cells. This is most likely caused by a cytotoxic effect of the 25 kDa PEI Lot#2 at the conditions used in the Karste *et al*. protocol, which was not seen for the 25 kDa PEI Lot#1.

In comparison to 25 kDa PEI, the linear 40 kDa PEI used according to the Karste *et al*. protocol instantly yielded the second highest eGFP mean value, despite the suboptimal fraction of gated living cells of 60%. Apparently, both described methods have been individually optimized for the specific 25 kDa PEI Lot. The linear 40 kDa PEI seems to be a promising alternative, which needs further optimization and evaluation of the protocol to establish reproducible conditions for TGE.

### Analysing the differences between 25 kDa and 40 kDa PEI Lots

**Fig 2:**
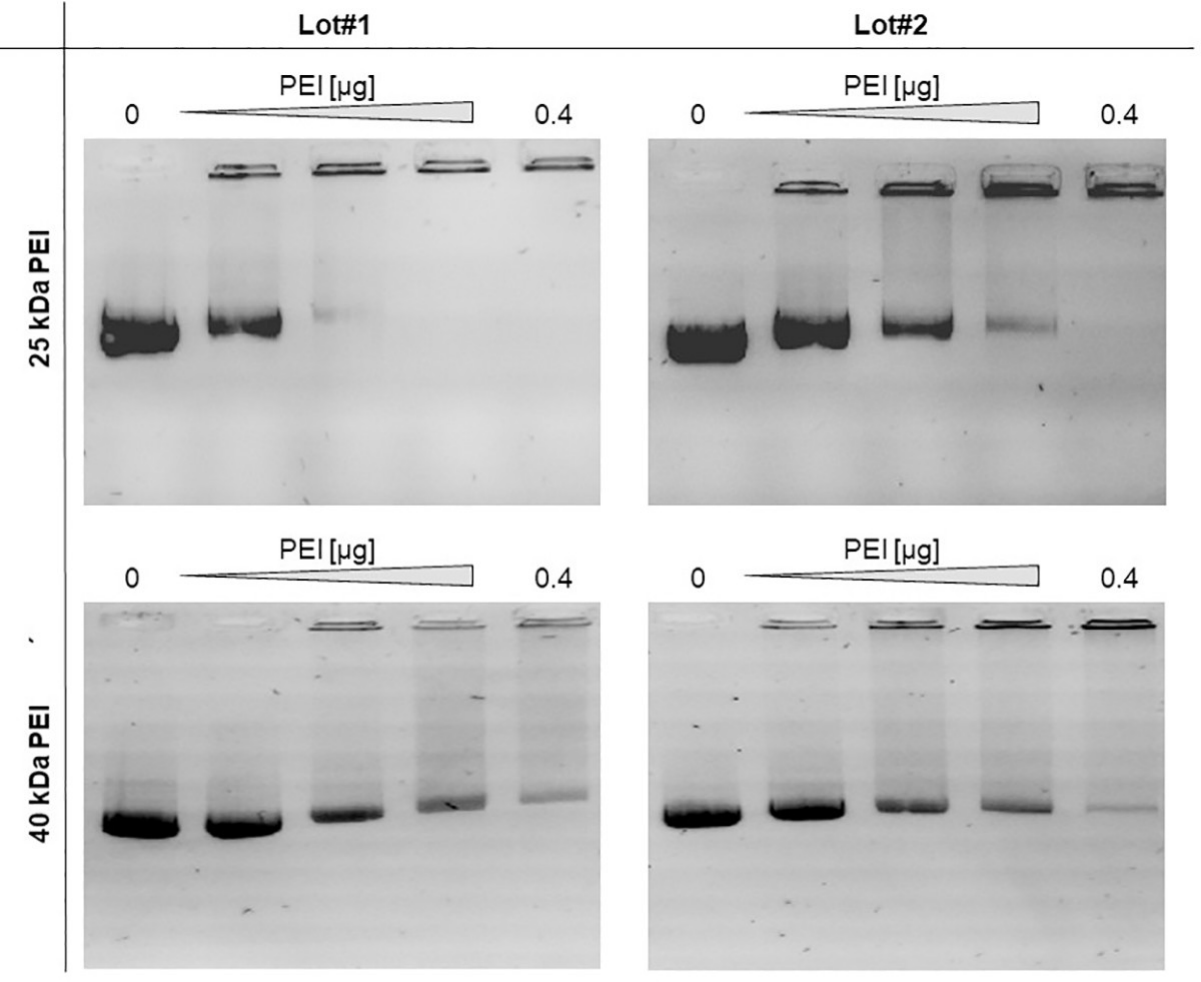
DNA agarose gel migration assay. DNA condensation ability of the different PEI 25 kDa and 40 kDa Lots was analysed by DNA gel retardation. 1 μg plasmid DNA was incubated at RT with the respective PEI amount (0 μg, 0.1 μg, 0.2 μg, 0.3 μg, 0.4 μg). The migration behaviour of the DNA:PEI complex by agarose gel electrophoresis was observed.

Obviously, the utilization of different PEI Lots leads to variations in transfection efficiencies. Therefore, the PEI Lots must have different characteristics. One distinction could be a difference in binding affinity to DNA. The difference in migration of the bound and non-bound DNA (Fig 2) was tested by agarose gel electrophoresis as described by Delafosse [40]. Positively charged PEI masks the negative charge of the DNA, thereby hampering the migration of PEI bound DNA compared to unbound plasmid DNA. Notably, for the 25 kDa PEI Lot#2, which showed the poorest transfection efficiency using the Karste *et al*. protocol, the highest DNA binding affinity could be shown (almost complete inhibition of migration at a concentration of 0.2 μg PEI). In comparison, the 25 kDa PEI Lot#1, 40 kDa PEI Lot#1 and 40 kDa PEI Lot#2 did only show inhibition of migration of the plasmid DNA at a concentration of 0.4 μg PEI. A higher binding capacity may lead to a slow release of the plasmid DNA inside the cell and thereby results in the observed reduction of the transfection efficacy and GFP production (GFP mean value). Indeed, there are some studies indicating that the formation of weaker DNA/PEI complexes results in more efficient intracellular polyplex unpacking and thereby facilitates entry of the plasmid into the nucleus and subsequent DNA transcription [43, 45, 48].

### Establishing a robust protocol using linear 40 kDa PEI

**Fig 3:**
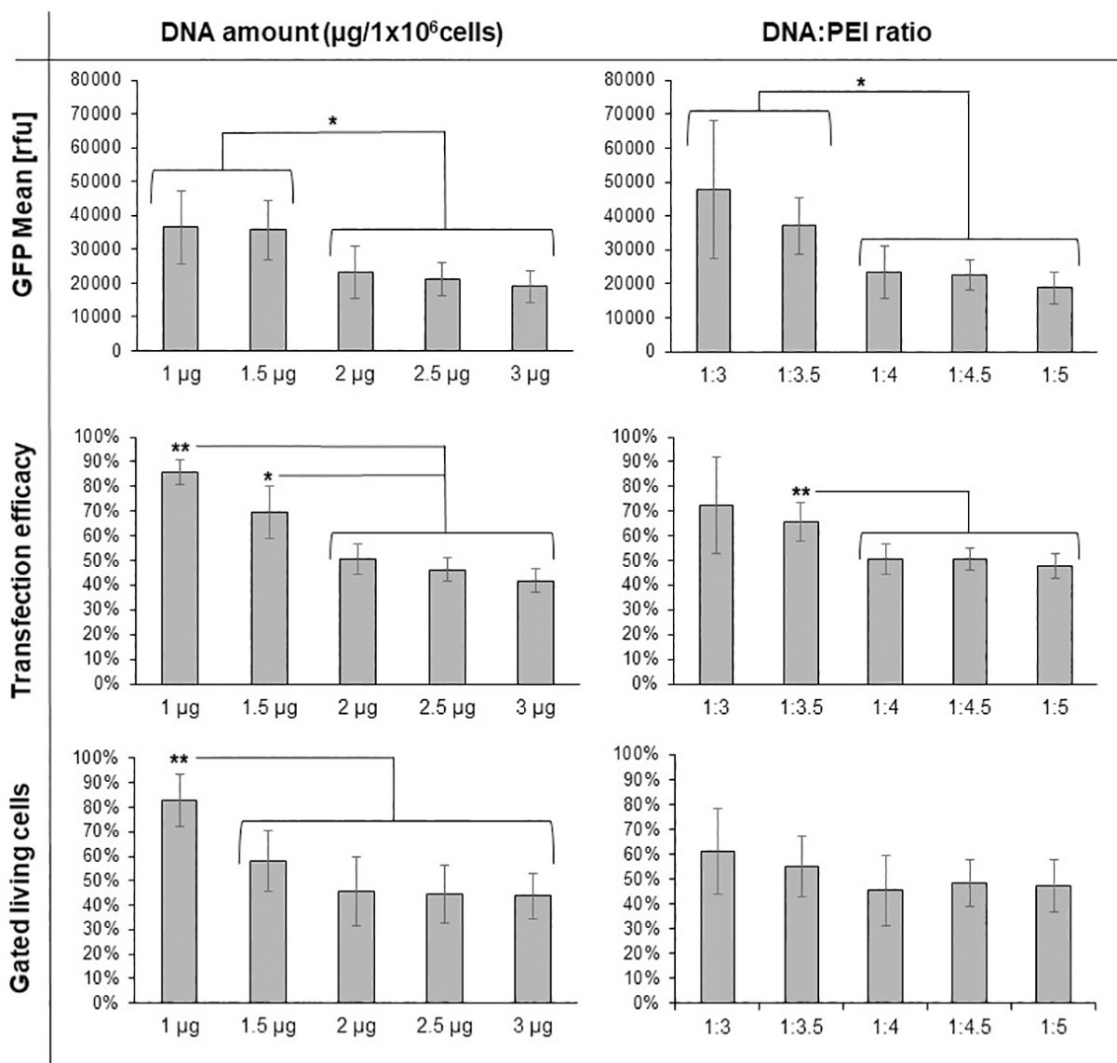
First round of TGE optimization using 40 kDa PEI. The parameters were varied starting from the conditions as described in Karste *et al*. (2 μg DNA/ 10^6^ cells, DNA:PEI ratio 1:4, cell culture density of 4x10^6^ c/mL during the initial 3 h of transfection). In each experiment only one parameter was varied: Either the total DNA amount (left) or the DNA:PEI ratio (right) was modified. The GFP mean value, the transfection efficacy and the fraction of gated living cells were measured. Every experiment was repeated four times; the resulting standard deviation is shown. A p value is indicated for individually compared conditions: p < 0.05 is indicated by **, a p value < 0.1 is indicated by *. A High Five cell line passaged over 180 times without freezing was used for all experiments.

To improve the reliability of the transient gene expression in High Five cells using 40 kDa PEI, we identified in a first round of optimization the best DNA:PEI ratio and the optimal DNA amounts for linear 40 kDa PEI Lot#1 (Fig 3), starting from the published protocol for linear 25 kDa PEI [34]. We initially varied the total DNA amount while keeping the DNA:PEI ratio constant at 1:4 and subsequently varied the DNA:PEI ratio while keeping the amount of DNA at 2 μg. The optimal conditions for 40 kDa PEI differ from those of 25 kDa PEI. Instead of 2 μg DNA as used in Karste *et al*. [34], the highest GFP mean value and transfection efficacy were reached at 1 μg DNA for the constant DNA:PEI ratio of 1:4. However, at a at a constant DNA amount of 2 μg the highest measured values were observed at a DNA:PEI ratio of 1:3.

**Fig 4:**
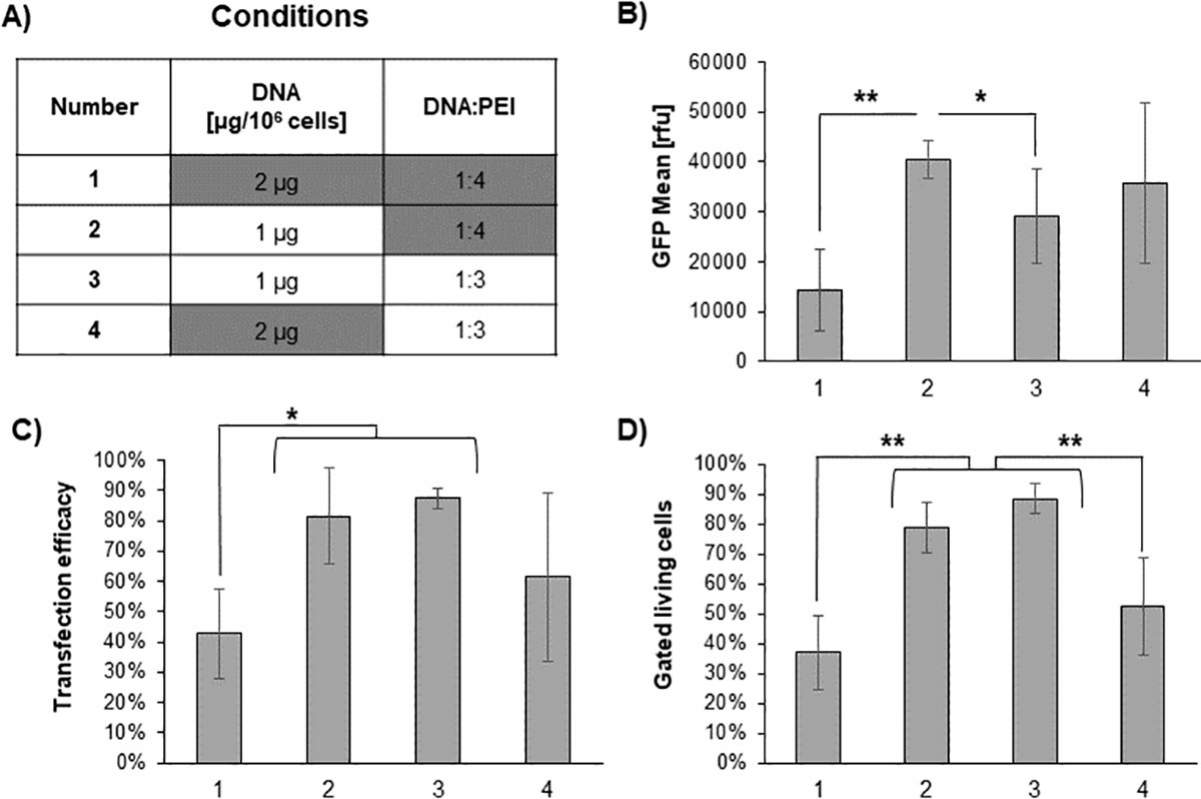
Second round of TGE optimization. A) The tested conditions represent a mixture of the best parameters found in the first round of optimization and the conditions described by Karste *et al*. (underlaid in grey). B) Shown is the GFP mean value [rfu], C) the ratio of transfected cells and D) the fraction of gated living cells for each condition (represented by number 1–4). Every experiment was repeated four times with the same cell line isolate with an identical passage number (over 180 passages); the resulting standard deviation is shown. A p value is indicated for individually compared conditions: p < 0.05 is indicated by **, a p value < 0.1 is indicated by *.

Therefore, we further compared the best performing conditions for the DNA:PEI ratio of 1:4 to 1:3 and the total DNA amount of 1 μg/ 10^6^ cells to 2 μg/ 10^6^ cells (Fig 4). This second round of optimization showed an overall optimum at 1 μg DNA and a DNA:PEI ratio of 1:4 (condition 2). Here, at a transfection rate of ~80% the GFP mean value of 40.000 rfu is close to the GFP mean value reached for the best conditions of the 25 kDa PEI, Lot#1 as described in Karste *et al*. [34] (see Fig 1).

**Fig 5:**
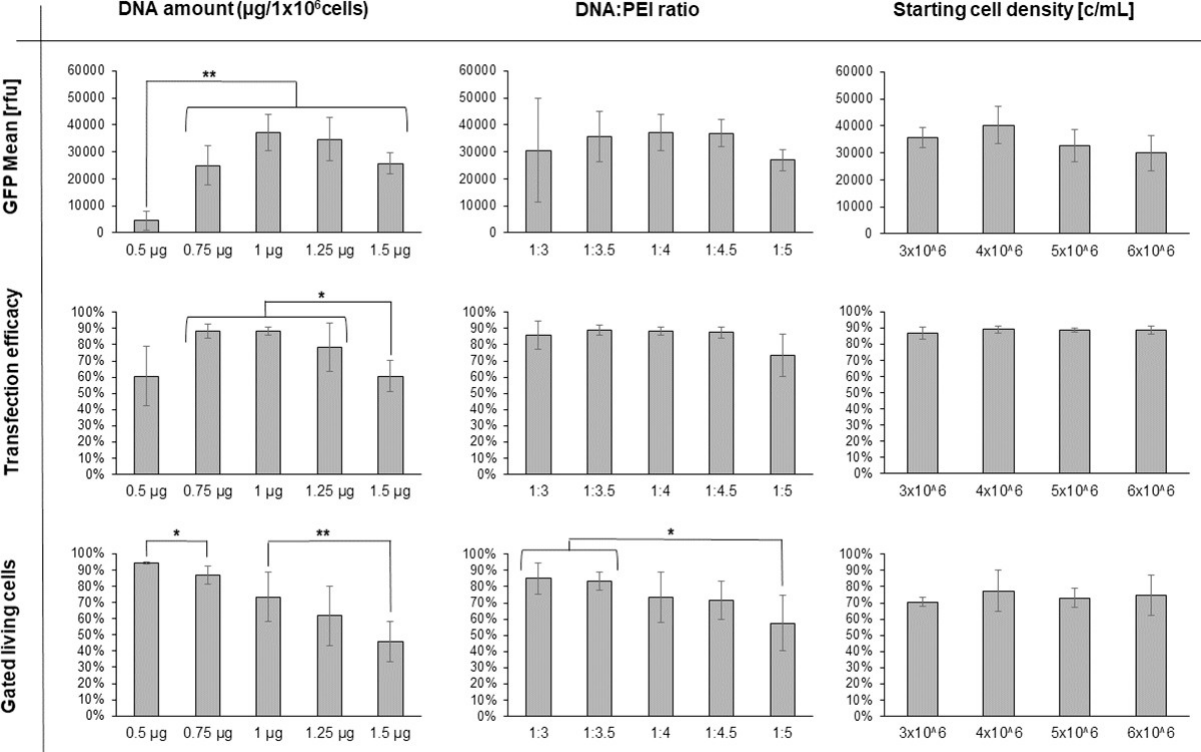
Final round of 40 kDa PEI optimization. Condition 2 (1 μg DNA/10^6^ cells, a DNA:PEI ratio of 1:4 and a cell culture density of 4x10^6^ c/mL during the first 3 h of transfection, determined in Fig 4) was used as starting point for further optimization. In each experiment only one parameter was varied: Either the total DNA amount, the DNA:PEI ratio or the cell culture density. The GFP mean value, the transfection efficacy as well as the fraction of gated living cells were determined. Every experiment was repeated four times; the resulting standard deviation is shown. A p value < 0.05 is indicated by **, a p value < 0.1 is indicated by *. A High Five cell line passaged over 180 times without freezing was used.

Condition 3 resulted in similar transfection efficiency and percentage of gated living cells as compared to condition 2. However, the GFP mean value was lower (p < 0.1) and a clear increase in standard deviation was observed. In comparison, for condition 4 a further increase in standard deviation was visible. Furthermore, a reduction in the vitality of the culture could be observed. In conclusion, the DNA:PEI ratio of 1:3 reduces the reproducibility of the experiments. Additionally, a lower amount of used DNA results in a reduction in cost for preparing the required amounts of DNA. Thus, condition 2 was used as a starting point to fine tune the optimal conditions in a final round of optimization. as well as examining the influence of the cell density on transfection efficiency during the first 3 h of the TGE (Fig 5).

During the final optimization the total DNA amount showed to have the greatest influence among all tested parameters. Reducing the amount of DNA below 1 μg of DNA/10^6^ cells improved the viability of the cells, but also decreased the eGFP yield. Increasing the amount of DNA to more than 1 μg of DNA/10^6^ cells the eGFP mean value, decreased the transfection rate as well as the viability, what may be due to increasing amounts of PEI negatively influencing cell viability.

The DNA:PEI ratio of 1:5 showed a significant impact on viability, which decreased probably due to the higher amount of 40kDa PEI. At the ratio of 1:3 the standard deviation of the GFP mean value substantial increased as shown before (Fig 4). Therefore, an optimal range in the DNA:PEI ratio was determined to be between 1:3.5 and 1:4.5.

In this round of optimization, the best cell density during the 3 h high cell density transfection step was additionally investigated. Only minor differences could be observed for cell densities between 3x10^6^ cells/mL up to 6x10^6^ cells/mL, with a slight preference for 4x10^6^ cells/mL.

In summary, the optimal condition using 40 kDa PEI was in a range of 1–1.25 μg of DNA/10^6^ cells, a DNA:PEI ratio of 1:3.5–1–4.5 and a cell density of 3 up to 5x10^6^ cells/mL based on high GFP mean value and high reproducibility (low standard deviation). For all further experiments we used 1 μg of DNA/10^6^ cells, a DNA:PEI ratio of 1:4 with a cell density of 4x10^6^ cells/mL during the first 3 h of transfection.

### Influence of PEI storage on transfection efficiency

The efficacy of PEI-based transfection is reported to be affected by numerous factors. Next to the parameters we previously analysed, the storage temperature of PEI solutions was reported to influence transfection efficacy [49].

**Fig 6:**
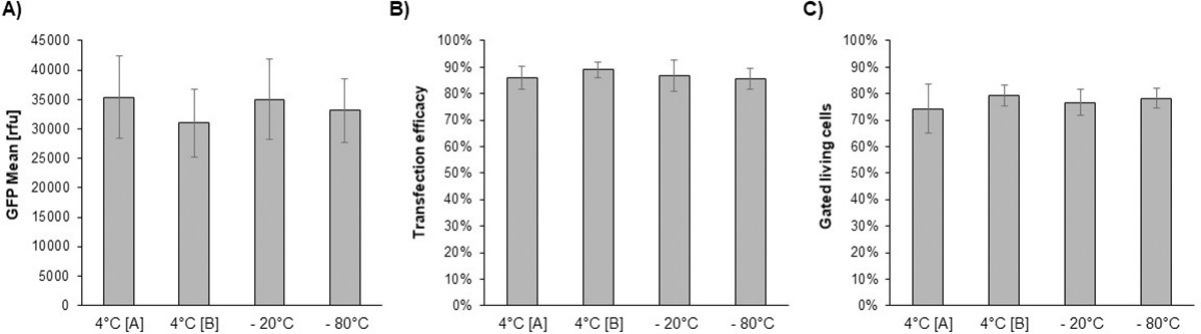
Influence of different storage temperatures and storage time of 40 kDa PEI on the eGFP expression levels. Batch [A] was stored over six months at 4°C whereas batch [B] was freshly prepared and stored max. three weeks at 4°C. Shown are A) the GFP mean value, B) the transfection efficacy and C) the fraction of gated living cells. A cell line with over 220 passages was used for this experiments and experiments were repeated four times.

Thus, transfection experiments with 1 mg/mL PEI solutions stored at different conditions (4°C, - 20°C or - 80°C) were performed (Fig 6). Only marginal differences of the eGFP levels if stored at - 20°C or - 80°C were observed. In parallel, the stability of PEI 40 kDa at 4°C was measured by comparing a PEI stock, which was stored for six months [A] to a freshly prepared stock [B]. Again, no significant difference was detected, concluding that neither the examined time nor the temperature of 40 kDa PEI storage did affect the expression level. The long-term stability of 40 kDa PEI further adds up to the robustness of the method.

### Influence of the vitality of the cell culture and passage number on transfection

During our experiments the vitality and growth performance of the High Five culture showed a huge influence on the transfection efficacy, GFP mean value as well as on the reproducibility of TGE.

**Fig 7:**
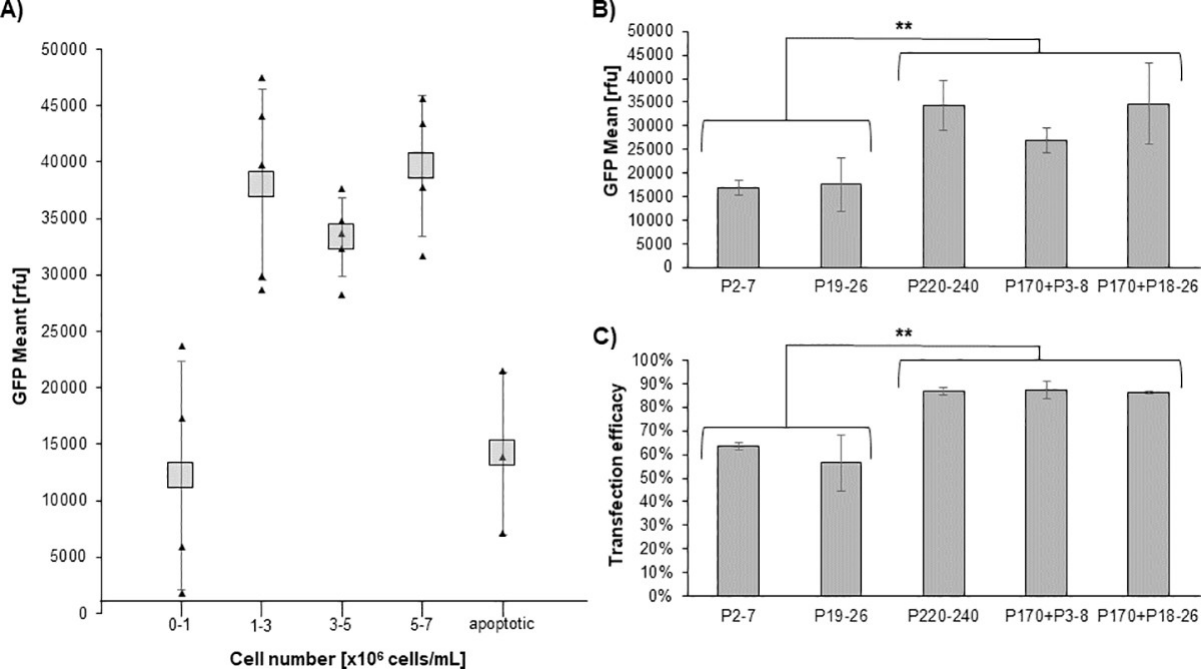
Influence of growth phase and passage number on GFP mean value. The experiment was done according to the optimised protocol. For all experiments a High Five cell line with over 200 passages was used. A) Influence of the cell density of the preculture (growth phase of the culture) on the GFP mean value. B) and C) comparison of the GFP mean value and transfection efficacy respectively of cell cultures of the derived from the same High 5 cell line in four independent successive experiments with the indicated passage number.

First of all, cell cultures growing exponentially performed much better then cells still in the lag phase (cell number below 1x10^6^ cells/mL) (Fig 7A). Interestingly, a cell number near the stationary phase did only marginally decrease the results as long as the viability remained above 95%. However, if the cells had reached the late stationary phase (apoptotic) as they were not passaged for four days, the GFP mean value dropped significantly, as expected. In total, to gain optimal results the cell density of the pre-culture should be between 1-5x10^6^ cells/mL before transfection. Important to note is that before transfection the required number of cells for the TGE needs to be centrifuged and resuspended at 4x10^6^ cells/mL in fresh medium to ensure optimal nutrition during the transfection step.

Secondly, we observed that during our experiments the performance of the cells depended on the passage number. Low passage numbers resulted in a lower transfection efficiency and GFP expression. In Fig 7B and 7C TGE results of cells are depicted with either a low passage number (P2-7 and P19-26), cultures with a high passage number which were not cryo conserved(P220-240) or cultures with a high passage number which did undergo a cryo-conservation at passage 170. The cryo-conserved cells were tested directly after revitalization (P170 + P3-8) and after 2 months of cultivation (P170 + P18-26).

The differences in the GFP mean values observed for the cultures with a low passage number compared to those with a higher passage number were significant (p < 0.05). Cells with a passage number of above ~170 passages gained up to 2 times higher GFP expression levels then cell cultures with a low passage number (below 30). In addition, the transfection efficacy was also significantly higher in cells with a high passage number leading to ~25% more transfected cells.

Interestingly, after the cryo-conservation of the culture with a high passage number (P170) the cells tendentially produced less eGFP while the transfection rate remained similar to the continuously passaged culture (P220-240). However, this observation was not significant in T-test. Twenty passages after thawing, the revitalized culture showed no differences to the cell culture of passage 220–240. This indicates that at least part of the improvement over passage number is due to optimal adaptation to the environmental conditions which might be the result of genetic modifications.

**Fig 8:**
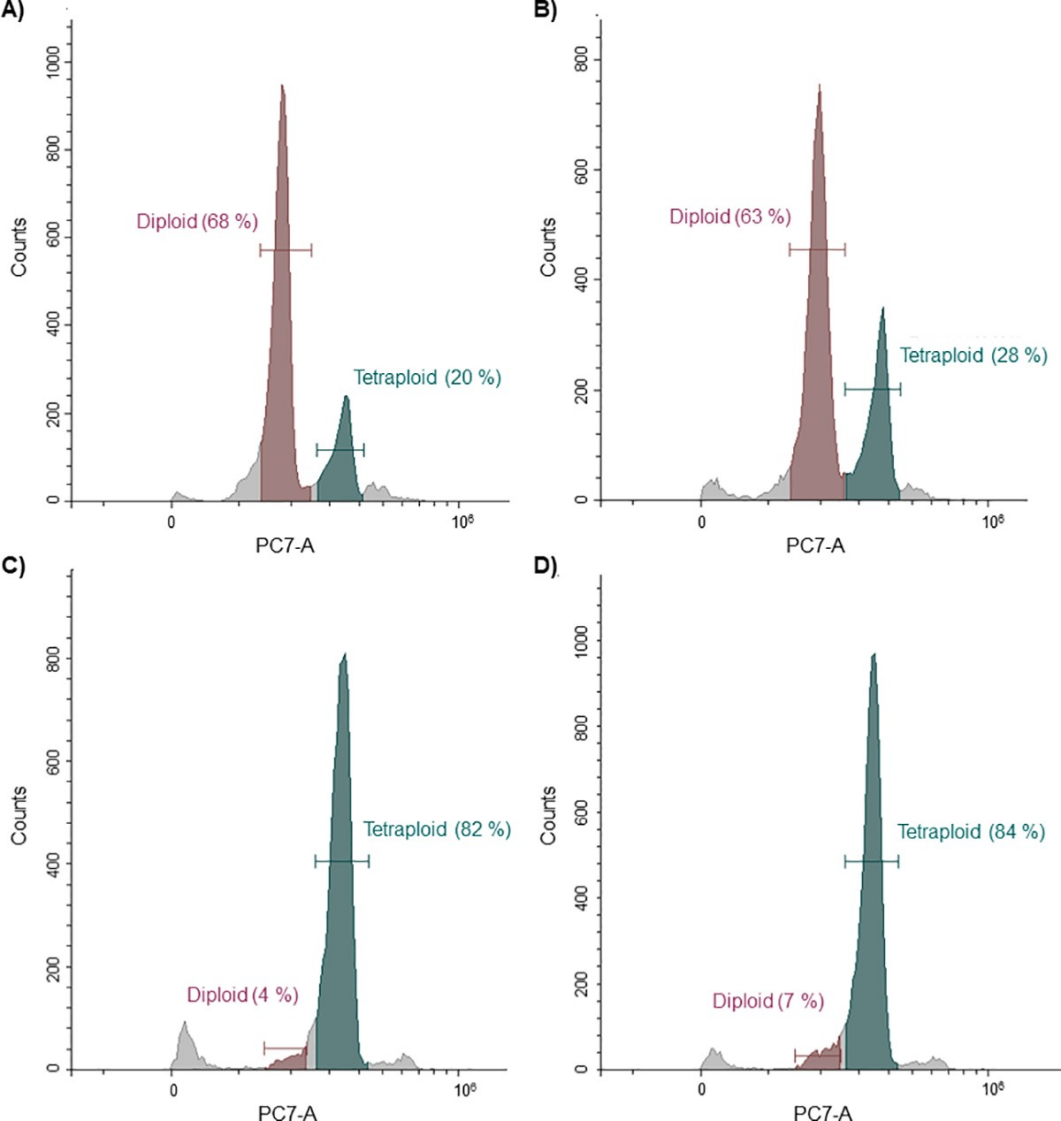
Polyploidy of a High Five cell line over a progressing passage number. Cells are shown after A) four passages, B) 20 passages, C) 220 passages and D) after 191 passages while frozen and thawed at passage 170. In each experiment the red fluorescence (PI counts) of 10.000 single cell events is depicted.

As described by Vaughn ([50] and Jarmann-Smith [42], lepidopteran cell lines can be mixoploid and cytogenetically instable. The change in ratio of basically diploid into mainly tetraploid cells with increasing culture passage number could be an explanation for the increased transfection efficiency and higher GFP expression level. To test this hypothesis, the DNA content of cells in cultures with different passage numbers was analysed by flow cytometer as described in Jarman-Smith [42]. Briefly, the nuclei of the cells were isolated, the DNA stained with propidium iodide (PI) and the PI fluorescence in the nuclei was measured in the cytometer (Fig 8).

The results clearly demonstrate an increase in the total amount of genomic DNA per cell in cultures with a higher passage number, which is probably caused by a shift from mainly diploid to an essentially tetraploid culture. This effect can already be observed after 20 passages, where the tetraploidy increases from ~20% to ~28%, while the number of diploid cells decreases from ~68% to ~63%. The DNA amount in the cells of the culture (P170 + P21) with one cryo-conservation cycle was comparable to the cells which were continuously passaged 220 times. In both cases most cells have become tetraploid and only a few cells remained diploid. In conclusion, tetraploidy is a likely explanation for the observed higher protein production and higher viability after the transfection of cells with high passage numbers. Thus, to gain higher and reproducible transfection levels tetraploidy of the used High Five cells should be confirmed by measuring the DNA amount per cell over several passages. After a tetraploid cell line is gained, a master cell bank can be constructed and used for further productions adding to reproducibly high TGE protein production levels.

### Testing the reproducibility of the new method

To validate and ensure the reproducibility of our optimized method a benchmark study with an additional 40 kDa PEI Lot#2, different High Five cell line isolates, different labs (RVZ Würzburg and HZI Braunschweig) and different cultivation media was done.

**Fig 9:**
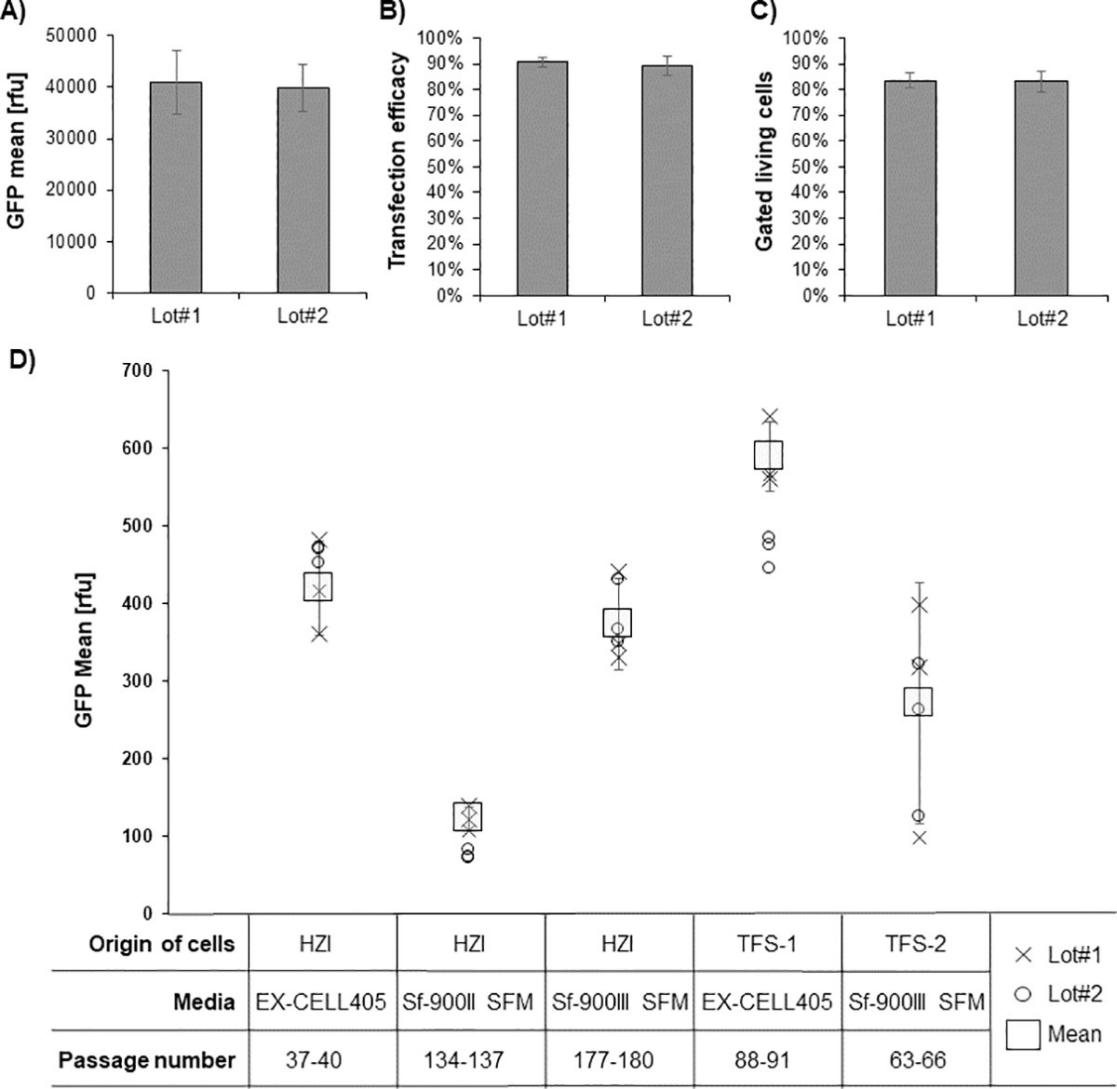
Comparison of the performance of two PEI 40 kDa batches, different medium and different cell lines in the optimized TGE method. A) GFP mean value, B) transfection efficacy and C) fraction of gated living cells of the cell cultures transfected in the RVZ using the two different PEI 40 kDa batches. A High Five cell line originating from the HZI with over 220 passages was used for this experiments and experiments were repeated four times. D) GFP mean value of different High Five cell lines adapted to different media using two different PEI 40 kDa batches. The GFP mean value was here measured at the HZI in the Guava (Merck Millipore), not in the Cytoflex (Beckmann Coulter) as before. Origin of cells, medium and passage number are indicated.

Fig 9A–9C shows a similar performance of the 40 kDa PEI Lots, indicating high reproducibility independent of the used PEI batch.

In addition, three High Five cell line isolates from different sources were compared in performance at the HZI in Braunschweig after adaptation to the respective culture medium for more than 3 months(Fig 9D). First of all, it was noted that the choice of medium significantly impacts the TGE. Here the Sf-900 II SFM medium clearly showed the lowest performance compared to EX-CELL 405 and Sf-900 III SFM medium. As in our protocol PEI and plasmid DNA are added directly to the medium without precomplexation, substances hampering the PEI-plasmid DNA complex formation in the Sf-900 II medium may be a plausible explanation.

As shown before, the use of both linear 40 kDa PEI batches (#Lot1 and #Lot2, Fig 9D) retained reproducibility of the individually tested combination of medium and High Five isolates on the GFP expression level.

In respect to the origin of the High Five clonal isolates, the TFS-1 cell line performed best. However, the HZI cell line (P37-40) may require longer passaging to reach optimal TGE efficiency. Interestingly, TFS-2 had a high standard deviation in GFP expression and did not perform as well as the HZI cell line in the Sf-900 III SFM medium even though both cell lines originate from the same Boyce-Thompson isolate. This may also be caused by incomplete adaption to the Sf-900 III SFM medium or an incomplete shift to tetraploidy.

In conclusion, to reproducibly implement this method and gain the same results, we recommend to use the High Five cell line isolate obtained from Thermo Fisher Scientific adapted to the EX-CELL 405 culture medium for more than 80 passages and additional testing for the tetraploidy of the cells.

### Recombinant protein production of HA1

**Fig 10:**
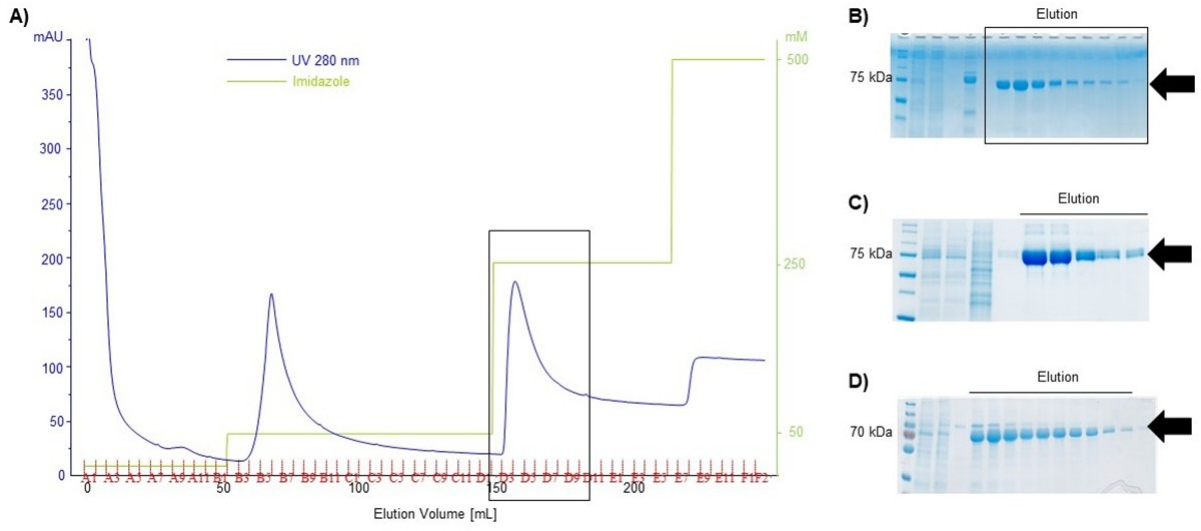
Expression and purification of HA1 (monomeric ~70 kDa) in High Five cells (HZI lab) using TGE and BEVS and TGE in HEK293-6E cells. A) Chromatogram of the HA1 affinity purification using the His tag, produced by TGE in High Five cells. After the washing step at 50 mM Imidazole the protein was eluted using 250 mM Imidazole (boxed area) B) Denaturing SDS gel analysis of the fractions of the eluted HA1 (High Five TGE) C) Denaturing SDS gel analysis of HA1 produced in HEK293-6E cells. D) HA1 produced in High Five cells using BEVS. The purification was done in all cases using the same procedure.

Finally, the performance in protein expression of the described plasmid-based High Five transient transfection system was compared to the well-known HEK293-6E expression system [36] (Fig 10). Both are fast and easy to handle plasmid-based eukaryotic expression systems. The optimization of our new protocol was done with a highly producible intracellular small protein (eGFP). Therefore, the comparison was done with the secreted trimeric HA1 (Hemagglutinin) as target protein. This is an important influenza virus glycoprotein used for vaccination strategies.

The yield (~13 mg/L) of purified HA1 protein was comparable in both High Five cells as well as in HEK 293-6E cells, whereas the yield of the BEVS in High Five cells was varying between 3–10 mg/mL. The lower yield in purified protein in the BEVS compared to TGE may be due to the loss of the performance of the secretion system of the cells during the late phase of the viral infection process. The observed high variability in yield was mainly caused by batch-to-batch variation in quality of virus stocks, which remains a major bottle-neck in the BEVS for large scale protein production.

Although robust expression of HA1 by TGE both in HEK 293-6E and High Five cells is feasible, the HA1 produced in HEK293-6E cells aggregated soon after protein purification. The aggregation of the HA1 produced in HEK293-6e could not be solved by screening for a stabilising buffer condition using the thermoflour assay. The HA1 produced in HEK 293-6E cells is post translationally modified and carries the complex mammalian glycosylation. In comparison, the insect cells produce proteins with shorter mannose-type glycans which allowed the HA1 protein to remain stable in solution and enabled 3 D structural analysis by X-ray crystallography. Thus, even though both expression hosts produced similar amounts of protein in this case the TGE insect cell system was favoured.

## Conclusion

In this study we tested our plasmid-based method published before [34] in regard of reproducibility and feasible implementation in different laboratories. The replacement of the linear 25 kDa PEI by linear 40 kDa PEI was the most important factor to circumvent the observed batch-to-batch variability in performance of different 25 kDa PEI Lots. So far, we could confirm that tested 40 kDa PEI Lots do not show relevant batch-to-batch variability for independently purchased lots. In addition, 40 kDa PEI performance was not influenced by different storage conditions and time of storage. Furthermore, we could successfully transfer and implement the described method to different laboratories by cultivating the commercially available High Five cell line (Thermo Fisher) over ~80 passages in EX-CELL 405 medium. The high passage number resulted in mainly tetraploid cells likely, explaining the increase in recombinant protein production. The cell density of the pre-culture was not influencing the reproducibility of the method, as long as exponential growth was maintained and viability was above 95%. In contrast, the choice of culture medium is important for high levels of transfection efficiency and protein production. Cells cultivated in EX-CELL 405 medium showed the best performance in TGE.

Important to note is that the choice and influence of the applied expression vector is a relevant factor for efficient TGE as shown before [37]. Furthermore, we did not pre-incubate PEI and DNA for complexation, but added subsequently DNA and PEI directly to the cell culture at a high cell density. This corresponds to the data published recently by Puente *et al*. [35], were the shortest tested pre-incubation of less than one minute gave the best results during TGE in High Five cells. Moreover, the best expression was also shown for high cell density transfection of HEK cells without pre complexing [51].

Finally, the applicability of virus-free transient transfection in High Five cells was underlined by gaining similar amounts of HA1 protein compared to TGE in HEK293-6E. Both methods even produced higher yields as in BEVS.

In conclusion, we improved robustness of the TGE in High Five cells and transferability of the method to other laboratories, where it can be used as a fast and simple expression system to produce high yields of target proteins.
